# Infarct growth rate predicts functional outcome after successful mechanical thrombectomy in patients with acute ischemic stroke

**DOI:** 10.3389/fneur.2026.1794343

**Published:** 2026-05-19

**Authors:** Wenhao Han, Chang Liu, Mengyu Ji, Jian Xue, Siping Guo, Bo Li, Yizhi Liu, Yonggang Hao

**Affiliations:** 1 Department of Neurology, The Fouth Affiliated Hospital of Soochow University, Suzhou, Jiangsu Province, China; 2 Department of Interventional Radiology, The First Affiliated Hospital of Soochow University, Suzhou, Jiangsu Province, China

**Keywords:** acute ischemic stroke, endovascular treatment, functional outcome, hypoperfusion intensity, infarct growth rate

## Abstract

**Background and purpose:**

Despite successful recanalization following endovascular mechanical thrombectomy (EVT) in patients with large-vessel occlusive stroke, a subset of patients continues to experience poor outcomes. This study aims to analyze the association between core infarct growth rate and patient prognosis, and to examine the interaction between infarct growth rate (IGR) and hypoperfusion intensity ratio (HIR).

**Methods:**

We retrospectively analyzed 142 patients with acute anterior circulation occlusion who achieved successful recanalization following EVT, defined as modified Thrombolysis in Cerebral Infarction (mTICI) grade 2b or 3. Patients were stratified into good (modified Rankin Scale [mRS] 0–2) and poor (mRS 3–6) prognosis groups based on 3-month mRS scores. IGR was calculated as the infarct core volume divided by the time from symptom onset to imaging. Infarct core volume was delineated as the volume of brain tissue with relative cerebral blood flow <30%, quantified using software based on perfusion imaging. HIR was defined as the ratio of tissue volume with T_max_ > 10 s to that with T_max_ > 6 s on CT perfusion imaging. Multivariate logistic regression was used to identify independent predictors of clinical outcome, and receiver operating characteristic curves were generated to evaluate their prognostic value.

**Results:**

Among the 142 patients, 91 achieved a good outcome (mRS 0–2), whereas 51 had a poor outcome (mRS 3–6). The cohort included 103 males (72.5%), with a median age of 68 (IQR: 58–74) and a median baseline NIHSS of 13 (IQR: 9–15). Multivariate analysis identified hypertension (odds ratio [OR] = 5.14, 95% CI: 1.15–22.87), baseline NIHSS score (OR = 1.25, 95% CI: 1.07–1.46), IGR (OR = 1.10, 95% CI: 1.01–1.21), HIR (OR = 380.71, 95% CI: 6.29–23,037.59), and cystatin C levels (OR = 26.65, 95% CI: 2.19–324.55) as independent predictors of poor outcome. HIR was the primary determinant of IGR, with higher HIR significantly associated with accelerated infarct growth (adjusted OR = 18.75, 95% CI: 2.45–143.54; *p* = 0.005).

**Conclusion:**

Hypertension, baseline NIHSS score, IGR, HIR, and cystatin C levels are independent predictors of poor functional outcome despite successful recanalization. Among these, HIR demonstrated strong discriminatory power in identifying patients with rapid versus slow infarct progression.

## Introduction

Endovascular mechanical thrombectomy (EVT) is an established treatment for acute ischemic stroke due to large-vessel occlusion, significantly improving reperfusion rates and clinical outcomes (1, 2). In recent years, advances in thrombectomy techniques have resulted in successful revascularization in more than 70% of cases; however, favorable functional outcomes are achieved in only about 50% of patients (3, 4). Poor prognosis has been associated with advanced age, high pre-treatment National Institutes of Health Stroke Scale (NIHSS) scores, hemorrhagic transformation, and failure of revascularization (5). Additionally, collateral circulation status and the infarct growth rate (IGR) are critical determinants of patient outcomes (6).

Patients with poor prognosis often exhibit rapid infarct core progression, with ischemic tissue rapidly converting to irreversible infarction, resulting in larger core volumes (7). Conversely, some patients maintain relatively small infarct cores even after prolonged onset-to-imaging intervals. The DEFUSE 3 and DAWN trials have expanded the therapeutic time window for endovascular treatment (EVT) in selected patients with acute anterior circulation ischemic stroke to 6–16 h and 24 h, respectively. However, the underlying pathophysiological mechanisms accounting for this extended time window remain incompletely elucidated. A slower IGR and robust collateral circulation may partly explain the rationale behind extending the EVT treatment window in these trials (8, 9). Early identification of this patient subgroup is essential for optimizing triage, expediting reperfusion, and improving clinical outcomes (10). Therefore, this study aims to analyze the correlation between the growth rate of core infarction and patient prognosis after successful recanalization, with a focus on the interaction between IGR and hypoperfusion intensity ratio (HIR).

## Methods

### Patient population

This retrospective study included patients with acute anterior circulation large vessel occlusion (LVO) admitted to the First Hospital of Soochow University and the Fourth Hospital of Soochow University between January 2021 and December 2024. Inclusion criteria were: (1) age ≥18 years; (2) pre-thrombectomy imaging with a standardized protocol, including non-contrast computed tomography (CT), CT perfusion (CTP), and CT angiography (CTA); (3) confirmed anterior circulation macrovascular occlusion on CTA (involving the internal carotid artery or M1/M2 segments of the middle cerebral artery, or tandem lesions); and (4) successful reperfusion following endovascular treatment (modified treatment in cerebral infarction [mTICI] grade 2b or 3) (11).

Exclusion criteria: Patients were excluded if they met any of the following criteria: (1) unsuccessful revascularization following EMT (defined as mTICI < 2b); (2) incomplete clinical data or loss to follow-up; (3) pre-existing disability prior to stroke onset (modified Rankin Scale [mRS] ≥ 2); or (4) posterior circulation infarction.

### Data collection

Baseline clinical data collected included sex, age, and comorbid conditions such as previous stroke, hypertension, diabetes mellitus, dyslipidemia, atrial fibrillation, and coronary artery disease. Additional variables recorded were stroke etiology as classified by the Trial of ORG 10172 in Acute Stroke Treatment (TOAST), the NIHSS score, Alberta Stroke Program Early CT Score (ASPECTS) on admission, and relevant imaging data from CTP studies.

### Imaging analysis

IGR was defined as the ratio of infarct core volume to the time from stroke onset to imaging acquisition. Infarct core volume was delineated as the volume of brain tissue with relative cerebral blood flow <30%, quantified using NeuBrainCARE (NBC) software based on perfusion imaging. HIR reflects the proportion of severely delayed arrival time in ischemic tissues, and studies have confirmed its efficacy as an indicator for evaluating collateral circulation (12). The metric is calculated as the ratio of the volume of tissue with T_max_ > 10 s to the volume with Tmax >6 s on CTP imaging (13). The clot burden score (CBS), based on CTA, can quantify the extent of intracranial thrombosis in patients with AIS. The total score ranges from 0 to 10, with lower scores indicating longer thrombus length and more distant thrombus location (14).

### Statistical analysis

Continuous variables with normal distribution were reported as mean ± standard deviation (mean ± SD) and compared using independent samples t-tests. Variables with skewed distributions were expressed as median and interquartile range [M(Q1, Q3)], with group comparisons made via the Mann–Whitney U test. Categorical variables were summarized as frequencies and percentages, and differences between groups were assessed using the Chi-square test or Fisher’s exact test, as appropriate. Variables with *p* < 0.05 in univariate analysis were included in multivariable logistic regression models. Regression results were presented as odds ratios (OR) with corresponding 95% confidence intervals (95% CI). Predictive performance was evaluated using receiver operating characteristic (ROC) curve analysis, and the area under the curve (AUC) was calculated. The Youden index is used to determine the optimal cutoff value for IGR prediction value. All statistical analyses were performed using SPSS Statistics version 27.0 (IBM Corp., Armonk, NY). All *p*-values were two-sided, with statistical significance set at *p* < 0.05.

### Study outcomes

The primary outcome was functional status at 90 days, assessed using the modified Rankin Scale. A favorable outcome was defined as an mRS score of 0–2, while an unfavorable outcome was defined as an mRS score of 3–6. Safety outcomes included symptomatic intracranial hemorrhage—defined as imaging-confirmed hemorrhage accompanied by a ≥ 4-point increase in the NIHSS score—and all-cause mortality at 90 days (15).

## Results

### Baseline characteristics and univariate analysis

We included 142 patients with anterior circulation LVO who achieved successful recanalization following EVT (Table 1). Of these, 91 patients had a favorable outcome (mRS 0–2), while 51 had an unfavorable outcome (mRS 3–6). Univariate analysis revealed that patients with unfavorable outcomes were older (median age 71 vs. 64 years; *p* = 0.004), had higher rates of hypertension (70.9% vs. 52.7%; *p* = 0.038) and atrial fibrillation (56.9% vs. 25.3%; *p* < 0.001), and included fewer males (59% vs. 80%; *p* = 0.006). Regarding stroke etiology, the unfavorable outcome group had a lower proportion of large artery atherosclerosis (41% vs. 67%; *p* = 0.013) and a higher proportion of cardioembolic strokes (53% vs. 30%; *p* = 0.013). Patients with unfavorable outcomes exhibited significantly higher baseline NIHSS scores (15.31 ± 4.90 vs. 10.35 ± 4.53, *p* < 0.001) and larger core volumes on CTP (30.50 [5.50, 53.70] vs. 4.35 [1.18, 14.78] mL, *p* < 0.001). Compared to those with favorable outcomes, these patients had higher HIR values (0.45 [0.37, 0.55] vs. 0.26 [0.09, 0.36], *p* < 0.001), faster IGR(13.53 [3.03, 23.61] vs. 1.37 [0.25, 4.56] mL/h, *p* < 0.001), greater thrombus burden (6.50 [5.00, 8.00] vs. 5.00[4.00, 7.00], *p* < 0.001), and a higher incidence of symptomatic intracranial hemorrhage (31.4% vs. 3.3%, *p* < 0.001).

**Table 1:** Comparison of demographics and clinical characteristics between good and poor clinical outcome groups.

Clinical characteristics	Good outcome; mRS (0–2); *N* = 91	Poor outcome; mRS (3–6); *N* = 51	*p* value
Age, y, median (IQR)	64(54, 72)	71(61, 77)	0.004
Male, n (%)	73(80.2%)	30(58.8%)	0.006
Hypertension, n (%)	48(52.7%)	36(70.9%)	0.038
Diabetes, n (%)	7(7.7%)	5(9.8%)	0.905
Atrial fibrillation, n (%)	23(25.3%)	29(56.9%)	<0.001
Coronary artery disease, n (%)	6(6.6%)	3(5.9%)	1.000
Prior stroke, n (%)	11(12.1%)	6(11.8%)	0.955
Stroke etiology, n (%)			0.013
LAA	60(66.7%)	21(41.2%)	
Cardioembolic	27(30%)	27(52.9%)	
Others	3(3.3%)	3(5.9%)	
IV tPA administered, n (%)	58(63.7%)	26(51.0%)	0.138
MAP, mmHg, mean ± SD	99.09 ± 16.00	102.59 ± 19.03	0.246
NIHSS score, mean ± SD	10.35 ± 4.53	15.31 ± 4.90	<0.001
Ischemic core volume, ml, median (IQR)	4.35(1.18, 14.78)	30.50(5.50, 53.70)	<0.001
HIR, median (IQR)	0.26(0.09, 0.36)	0.45(0.37, 0.55)	<0.001
CBS, median (IQR)	6.50(5.00, 8.00)	5.00(4.00, 7.00)	0.002
IGR, ml/h, median (IQR)	1.37(0.25, 4.56)	13.53(3.03, 23.61)	<0.001
General anesthesia, n (%)	30(33.0%)	20(39.2%)	0.455
Mismatch ratio, median (IQR)	15.65(3.45, 51.76)	4.50(2.80, 12.40)	0.002
ASPECTS, median (IQR)	9(8, 10)	9(8, 10)	0.335
A/G, median (IQR)	1.60(1.50, 1.90)	1.50(1.30, 1.70)	0.004
Cystatin C, mg/L, median (IQR)	0.91(0.82, 1.10)	1.04(0.90, 1.26)	0.004
HDL-C, mmol/L, median (IQR)	0.95(0.82, 1.15)	1.13(0.89, 1.30)	0.012
LDL, mmol/L, median (IQR)	2.37 ± 0.68	2.36 ± 0.78	0.871
N/L, median (IQR)	3.31(1.95, 6.83)	3.76(1.64, 8.19)	0.819
sICH, n (%)	3(3.3%)	16(31.4%)	<0.001

Categorical variables are expressed as n(%) and continuous variables as median (interquartile range). HIR, hypoperfusion intensity ratio; IGR, infarct growth rate; NIHSS, National Institutes of Health Stroke Scale. CBS, the clot burden score; mRS, modified Rankin Scale; tPA, tissue-type plasminogen activator. LAA, Large artery Atherosclerosis; ASPECTS, indicates Alberta Stroke Program Early CT Score; MAP, Mean Arterial Pressure; sICH, Symptomatic Intracerebral Hemorrhage; A/G, albumin/globulin; N/L, Neutrophil/Lymphocyte.

### Multivariate logistic regression model

Variables demonstrating significant difference in univariate analyses were entered into a multivariable logistic regression model to control for potential confounding factors. The results identified hypertension as an independent predictor of poor outcome (OR 5.14; 95% CI, 1.15–22.87; *p* = 0.032). Higher baseline NIHSS scores were also associated with unfavorable outcomes (OR 1.25; 95% CI, 1.07–1.46; *p* = 0.004). Additionally, HIR (OR 380.71; 95% CI, 6.29–23,037.59; *p* = 0.005), IGR (OR 1.10; 95% CI, 1.01–1.21; *p* = 0.040), sICH (OR 18.75; 95% CI, 2.45–143.54; *p* = 0.005), and cystatin C levels (OR 26.65; 95% CI, 2.19–324.55; *p* = 0.010) emerged as independent predictors of unfavorable outcomes (Table 2).

**Table 2:** Results of multifactor logistic regression analysis.

Factor	Good outcome; mRS (0–2); *N* = 91	Poor outcome; mRS (3–6); *N* = 51	*p* value	aOR 95%CI
Hypertension	48(52.7%)	36(70.9%)	0.032	5.14(1.15–22.87)
Atrial fibrillation	23(25.3%)	29(56.9%)	0.555	0.66(0.16–2.64)
Age	64(54, 72)	71(61, 77)	0.183	0.96(0.90–1.02)
NIHSS score	10.35 ± 4.53	15.31 ± 4.90	0.004	1.25(1.07–1.46)
HIR	0.26(0.09, 0.36)	0.45(0.37, 0.55)	0.005	380.71(6.29–23037.59)
IGR,ml/h	1.37(0.25, 4.56)	13.53(3.03, 23.61)	0.040	1.10(1.01–1.21)
sICH	3(3.3%)	16(31.4%)	0.005	18.75(2.45–143.54)
Cystatin C,mg/L	0.91(0.82, 1.10)	1.04(0.90, 1.26)	0.010	26.65(2.19–324.55)

Variables with *p* ≤ 0.05 in univariate analyses were considered during multivariate logistic regression modeling and were modeled using binary logistic regression. CI denotes confidence intervals; OR denotes ratio of ratios.

**Figure 1:**
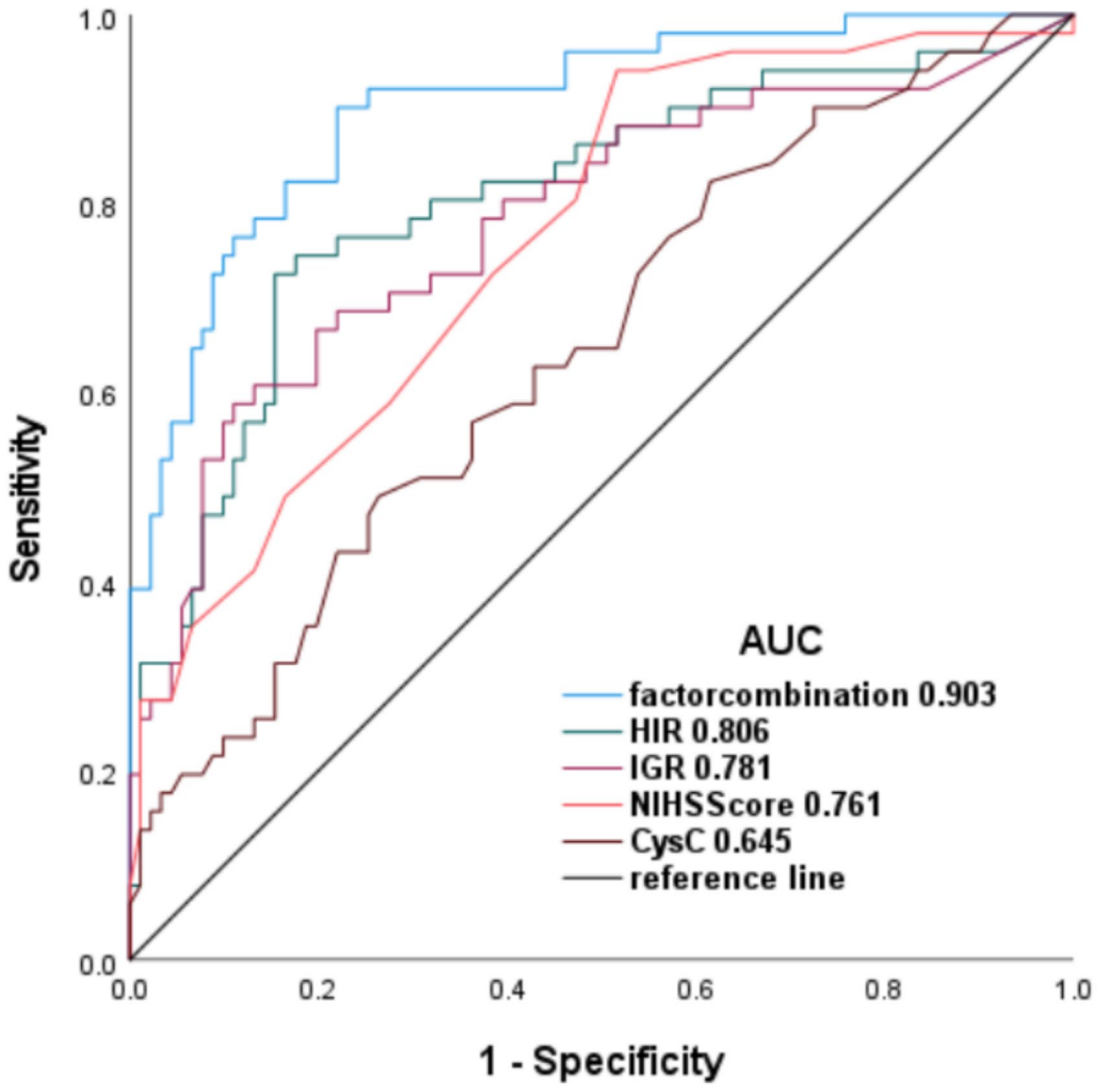
Receiver operating characteristics (ROC) analysis.

The optimal cutoff values for IGR and HIR to predict FR calculated using the Youden index were 11 mL/h and 0.40, respectively. The previously reported IGR threshold of 10 mL/h demonstrated higher specificity, while an HIR > 0.40 has been shown to more accurately reflect poor collateral status (7, 12, 16). After adjusting for confounders, multivariable logistic regression indicated that patients with HIR > 0.40 had significantly reduced odds of favorable outcomes compared to those with HIR < 0.40 (adjusted OR 7.49; 95% CI, 2.71–20.68; *p* < 0.001; Figure 1). Similarly, patients with IGR > 11 mL/h had markedly lower odds of achieving a favorable outcome compared to those with IGR ≤ 11 mL/h (adjusted OR 7.49; 95% CI, 2.71–20.68; *p* < 0.001; Table 3).

**Table 3:** Relationship between HIR and IGR optimal cutoff values and prognosis.

Factor	Good outcome mRS (0–2)	Poor outcome mRS (3–6)	*p* value	Adjusted OR 95%CI
	*N* = 91	*N* = 51		
HIR > 0.40	14(15.4%)	35(68.6%)	<0.001	7.49(2.71–20.68)
HIR < 0.40	77(84.6%)	16(31.4%)		
IGR > 11.04	9(9.9%)	31(60.8%)	<0.001	6.37(2.21–18.35)
IGR < 11.04	82(90.1%)	20(39.2%)		

Data were adjusted for age, sex, past history (Hypertension, Atrial fibrillation) and baseline NIHSS. CI indicates confidence interval; and OR, odds ratio.

### Predictive validity of clinical variables

ROC curve analysis was used to assess the predictive performance of individual risk factors. A multivariable model incorporating HIR, IGR, baseline NIHSS score, and cystatin C demonstrated excellent predictive accuracy (AUC 0.90; 95% CI, 0.85–0.95). Among individual predictors, HIR exhibited the highest discriminatory power (AUC 0.81; 95% CI, 0.73–0.89), followed by IGR (AUC 0.781; 95% CI, 0.697–0.865) and baseline NIHSS score (AUC 0.761; 95% CI, 0.681–0.842). Cystatin C showed moderate predictive ability (AUC 0.64; 95% CI, 0.55–0.74; Figure 1).

### Association between HIR and IGR

A higher HIR was significantly associated with faster IGR (*p* = 0.004). No statistically significant differences in IGR were observed between groups in the first two HIR quartiles (adjusted OR 3.42; 95% CI, 0.58–20.30; *p* = 0.175). However, patients with HIR > 0.45 had substantially increased odds of rapid infarct progression compared to those with HIR < 0.17 (adjusted OR 18.75; 95% CI, 2.45–143.54; *p* = 0.005), as determined by multivariable logistic regression controlling for confounders (Table 4).

**Table 4:** Association between HIR and IGR.

Factor	IGR < 11.04	IGR > 11.04	*p* value	Adjusted OR 95%CI
	*N* = 102	*N* = 40		
HIR < 0.17	34(33.3%)	2(5.0%)	0.004	
HIR: 0.17–0.33	29(28.4%)	5(12.5%)	0.175	3.42(0.58–20.30)
HIR: 0.33–0.45	23(22.5%)	12(30.0%)	0.024	8.29(1.53–44.93)
HIR: >0.45	16(15.7%)	21(52.5%)	0.001	15.74(3.02–82.00)

Data were adjusted for age, sex, past history (Hypertension, Atrial fibrillation) and baseline NIHSS. CI indicates confidence interval; and OR, odds ratio.

## Discussion

Our findings indicate that despite successful revascularization in patients with large-vessel occlusion, approximately one-third still experience poor outcomes. Baseline NIHSS scores, IGR, and HIR were strongly associated with functional outcomes following EVT. In addition, comorbid hypertension, symptomatic intracranial hemorrhage, and elevated cystatin C levels emerged as independent predictors of poor prognosis after mechanical thrombectomy.

Consistent with prior studies, we confirmed that high baseline NIHSS scores and post-procedural hemorrhagic transformation are strongly associated with unfavorable outcomes (7). Hypertension was identified as an independent predictor of poor prognosis, potentially due to its contribution to atherosclerosis, which may predispose patients to both large- and small-vessel occlusions (17). Previous studies have shown that chronic hypertension induces structural vascular remodeling, luminal narrowing, a reduction in collateral vessel number, endothelial damage, and impaired vasodilatory function (18, 19). In patients with comorbid hypertension who experience large-vessel occlusive stroke, compromised collateral circulation due to reduced collateral flow may limit compensation to the infarct core, contributing to poor clinical outcomes. Symptomatic intracranial hemorrhage is one of the severe complications following (EVT). Its core pathological mechanism involves severe disruption of the blood–brain barrier (BBB) and increased permeability in ischemic brain tissue after reperfusion, leading to rapid deterioration of neurological function and significantly offsetting the benefits of vascular recanalization (20). Furthermore, studies have demonstrated that intraventricular extension of the hematoma following intracerebral hemorrhage in patients with AIS is also an independent risk factor affecting patient prognosis (21). Our intergroup univariate analysis revealed that patients with LAA etiology had significantly higher rates of favorable outcomes compared to those with CE stroke. This observation aligns with recent evidence suggesting differential responses to adjunctive antiplatelet therapy based on stroke etiology. In a *post hoc* analysis of the RESCUE BT trial, Yue et al. found that among patients achieving ideal reperfusion (eTICI = 3), tirofiban significantly improved outcomes in the LAA subgroup with high baseline NIHSS scores (>13) (adjusted OR 4.67; 95% CI: 1.54–14.12; *p* < 0.05), whereas no such benefit was observed in CE patients (22). Arboix et al. further commented that this differential efficacy may be attributable to distinct pathophysiological mechanisms: thrombi in LAA stroke are typically more stable, allowing tirofiban to improve microcirculation, while CE patients with softer cardiac thrombi and poorer collateral circulation may experience limited recovery (23).

Our study identified serum cystatin C levels as an independent predictor of poor prognosis following endovascular therapy (EVT). Cystatin C, a lysosomal cysteine protease inhibitor present in all nucleated cells, serves as a sensitive biomarker for early kidney injury (24). A recent study similarly reported that elevated serum cystatin C levels independently predicted unfavorable outcomes, even in patients with successful recanalization, consistent with our findings (25). This may be attributed to the fact that cystatin C, as an inflammatory mediator, participates in the inflammatory cascade triggered by reperfusion following acute ischemic stroke, thereby exacerbating post-ischemic reperfusion injury (26). Furthermore, as a cysteine protease inhibitor, cystatin C can interact with proteases to maintain the dynamic balance between extracellular matrix deposition and degradation in the arterial intima (27). When the aforementioned balance is disrupted, compensatorily elevated cystatin C may be involved in the pathophysiological processes of atherosclerosis and plaque instability (28). This elevation in cystatin C levels may represent a compensatory response to ischemic injury. In coronary artery studies, elevated serum cystatin C levels have been demonstrated to predict no reflow phenomenon following percutaneous coronary intervention in patients with ST-segment elevation myocardial infarction (29). These findings suggest that cystatin C may be a promising therapeutic target, but further research is needed to determine its impact on infarct core size and long-term outcomes. Furthermore, the specific pathophysiological mechanisms by which cystatin C participates in the inflammatory cascade during ischemia–reperfusion injury remain unclear. Future studies should investigate the dynamic changes of cystatin C in the inflammatory response and its causal relationship with blood–brain barrier disruption, thereby providing a theoretical basis for developing anti-inflammatory therapeutic strategies targeting cystatin C and its related pathways.

Both HIR and IGR were also identified as independent predictors of poor prognosis. Essentially, IGR reflects the transition rate from reversible ischemic penumbra to irreversible core infarction (30). Previous studies defining IGR < 10 mL/h as slow progression patients revealed significantly higher rates of favorable outcomes after EVT compared to IGR > 10 mL/h fast progression patients. Further analysis demonstrated that among fast progression patients, those receiving EVT 6 h after onset had poorer prognoses than those treated within 6 h. These findings suggest that shortening the time window from onset to reperfusion is critical for improving outcomes in fast progression patients (7). Additionally, in studies involving patients with prolonged treatment time windows, both the DAWN study and defuse3 study established upper limits for core infarct volume (DAWN ≤51 mL, defuse3 ≤ 70 mL), which may suggest that the potential benefits obtained by patients with extended treatment windows in these studies were correlated with the rate of infarct progression (8, 9). Studies have shown that collateral circulation is a key factor determining the progression rate of infarction (31, 32). The collateral circulation can compensate for cerebral blood flow in the occluded vascular supply area, delaying the progression of ischemic tissue from the penumbra to the core infarct. This prolongs the therapeutic window, reduces the final infarct volume, and lowers the risk of hemorrhagic transformation (33). Previous studies have shown that ischemic preadaptation can enhance cerebral perfusion and reduce the recurrence of transient ischemic attacks in patients with intracranial arterial stenosis (34). This may be due to chronic hypoxia-ischemia promoting the development of functional collateral pathways and the maturation of preexisting vascular branches (35). These observations suggest that ischemic preconditioning may represent a novel adjunctive strategy to improve outcomes following endovascular thrombectomy (EVT). Nonetheless, further research is warranted to elucidate its role in collateral formation and long-term prognosis in stroke patients.

HIR can be directly obtained based on CTP, with a value of HIR > 0.4 defined as collateral circulation insufficiency (6). The Tmax parameter on CTP imaging can quantify the degree and volume of tissue hypoperfusion. Tmax refers to the time it takes for local brain tissue to reach peak contrast agent concentration after injection. A longer time delay indicates more severe hypoperfusion. Studies have found that lesions with Tmax> 6 s encompass varying degrees of hypoperfusion, including those with Tmax> 8 s and Tmax> 10 s (36). To differentiate the heterogeneity of varying hypoperfusion levels in ischemic tissues, Olivot et al. proposed the concept of volume ratio between severely hypoperfused areas and hypoperfused areas, defining the ratio of volumes in regions with Tmax> 10s to those with Tmax> 6 s as HIR. They found that HIR could serve as a predictive indicator for collateral circulation and also demonstrated correlations between HIR and infarction progression rate as well as patient clinical outcomes (6). Currently, HIR has been confirmed by multiple studies as a predictive indicator of collateral circulation. The higher the HIR value, the poorer the collateral circulation in patients (12, 37). Moreover, previous studies comparing intraoperative ASITN/SIR collateral scores assessed in patients with HIR values derived from CTP/MRP analysis revealed a significant correlation between HIR and favorable ASITN/SIR collateral scores. Furthermore, a HIR value <0.4 was identified as the optimal cutoff for predicting good collateral circulation (ASITN/SIR score of 3–4) (12). This study revealed that a HIR value <0.4 not only predicts favorable collateral circulation but also serves as an independent risk factor for poor prognosis when HIR > 0.4. Compared to traditional ASITN/SIR scores based on DSA evaluation, HIR values directly calculated by automated software effectively reduce subjective bias in assessment. Further analysis demonstrated a strong correlation between IGR and HIR, with HIR being the independent risk factor for accelerated IGR progression. These findings align with previous research examining factors influencing core infarction progression in transferred patients, which identified HIR > 0.4 as the optimal imaging marker for predicting core infarction progression during hospital transfer (38). Another study confirmed that HIR was a major predictor of rapid core infarct progression in AIS-LVO patients, whether assessed based on CT perfusion imaging or MR perfusion imaging (39). In summary, HIR as a directly measurable parameter for evaluating collateral circulation in CTP imaging, holds significant value in identifying patients’ collateral circulation status. For patients with an HIR > 0.4 postoperatively, active assessment of neurological function should be conducted to minimize the risk of deterioration.

Preoperative identification of high-risk factors remains essential for guiding EVT strategies. In such patients, minimizing the time to reperfusion is critical to salvaging the ischemic penumbra, limiting infarct core expansion, and optimizing clinical outcomes. Currently, the threshold for defining a fast IGR remains inconsistent across studies, with reported cutoff values for identifying fast progressors ranging from 4.8 mL/h to 25 mL/h (40, 41). Future multicenter studies should be conducted to standardize the calculation process and cutoff value criteria for IGR, and develop a multivariate prediction model to enhance the feasibility of clinical operations. Furthermore, due to the limitations inherent to a retrospective study design, we did not include the onset-to-imaging time (OIT) or the imaging-to-recanalization time (IRT) in the multivariate analysis. Theoretically, a longer OIT may be associated with a larger infarct core volume, and the calculation of IGR could be influenced by non-linear infarct growth. Meanwhile, IRT directly affects the extent of salvageable ischemic penumbra. Although we calculated IGR as the infarct core volume divided by OIT, the clinical significance of the same IGR value may differ across different OIT time windows. Future studies should further investigate the interaction between OIT, IRT, and IGR to optimize individualized revascularization strategies.

This study has several limitations. First, post-awakening strokes were not excluded, and IGR was calculated based on the patient’s last known well time rather than the actual time of symptom onset, potentially reducing accuracy. Second, infarct growth is unlikely to be linear; thus, estimates based solely on admission imaging may be imprecise. Third, the study did not assess the impact of different onset-to-reperfusion time windows on outcomes, and the prognostic implications of recanalization timing across varying IGR profiles remain unaddressed. Fourth, HIR was used as a surrogate marker for collateral circulation in this study. Although previous studies have confirmed that HIR correlates well with the ASITN/SIR grading system, and that an HIR < 0.4 has high sensitivity and specificity for predicting good collateral status, HIR cannot fully replace direct angiographic assessment (42). A comprehensive evaluation integrating HIR with ASITN/SIR grading remains necessary. Given that intraoperative DSA collateral grading was not routinely collected in this study, our analysis of collateral status may lack precision.

## Conclusion

Our findings indicate that hypertension, baseline NIHSS score, IGR, HIR, and cystatin C levels are key determinants of clinical outcomes in EVT patients. Among these, HIR emerged as a robust imaging marker of collateral status and a significant predictor of IGR.

## Data Availability

The data analyzed in this study is subject to the following licenses/restrictions: the data in this study contains clinical privacy information of human subjects. The use of such data has been approved by the [Ethics Committee of the First Affiliated Hospital of Soochow University and the Fourth Affiliated Hospital of Soochow University]. The approval stipulates that raw data must not be disclosed or disseminated externally to protect the privacy and rights of the subjects. Requests to access these datasets should be directed to Wenhao Han, hwh202399@163.com.
